# Intramedullary fixation versus plate fixation for displaced mid-shaft clavicle fractures: A systematic review of overlapping meta-analyses

**DOI:** 10.1097/MD.0000000000009752

**Published:** 2018-01-26

**Authors:** Lin Xie, Zhigang Zhao, Shujun Zhang, Yabin Hu

**Affiliations:** a Department of Orthopedic Surgery, Wuhan Orthopedic Hospital, Wuhan Puai Hospital, Huazhong University of Science and Technology, Wuhan; b Department of Orthopedic Surgery, The Sixth Affiliated Hospital, Xinjiang Medical University, Urumqi, Xinjiang Province, China.

**Keywords:** displaced mid-shaft clavicle fractures (DMCFs), intramedullary fixation (IMF), overlapping meta-analyses, plate fixation (PF)

## Abstract

**Background:**

Displaced mid-shaft clavicle fractures (DMCFs) are common injuries. Both intramedullary fixation (IMF) and plate fixation (PF) have been described and routinely used. Multiple trials have been conducted to compare these treatments. Multiple meta-analyses have been published to compare IMF and PF treatment for DMCFs; however, the results remain controversial. The purposes of this study were to perform a systematic review of overlapping meta-analyses comparing IMF and PF treatment for DMCFs, to help decision makers critically evaluate the current meta-analyses, and to propose a guide through the best available evidence.

**Method:**

We searched the Cochrane library, PubMed, and EMBASE data bases. Two authors independently scanned titles and abstracts to exclude irrelevant articles and identify meta-analyses that met the eligibility criteria. The methodological quality of the meta-analysis was independently assessed by the 2 authors using the Oxford Centre for Evidence-based Medicine Levels of Evidence and the Assessment of Multiple Systematic Reviews (AMSTAR) tool. Heterogeneity information of each variable was extracted from the included studies. An *I*^2^ of <60% is accepted in this systematic review. The Jadad algorithm was then applied to determine which of the meta-analyses provided the best evidence.

**Results:**

Eight meta-analysis met the inclusion criteria in this study. AMSTAR scores varied from 7 to 9. Heterogeneity of each outcome was acceptable. Four authors independently selected the same meta-analysis as providing the highest quality of evidence using the Jadad decision algorithm.

**Conclusion:**

This systematic review of overlapping meta-analyses suggests that compared with PF, major reintervention and refracture after implant removal occurred more frequently after PF of DMCFs. No differences in terms of function and non-union between PF and IMF were observed. Future research should focus on fracture selection for IMF and further improvement of plates and IM devices.

## 1 Introduction

Displaced mid-shaft clavicle fractures (DMCFs) are common fractures of upper limb accounting for 2.6% to 4% of all fractures and its incidence increased from 35.6 per 100,000 person-years in 2001 to 59.3 per 100,000 person-years in 2012.^[1]^ The clavicle is an S-shaped bone connecting the trunk of the body to the arm which is also a symbol of beautiful body. Most clavicular fractures occur in the mid-shaft and most of mid-shaft clavicular fractures are displaced.^[1]^ In general, DMCFs occur in young men and are largely caused by falls, motor vehicle accidents as well as sporting injuries.

Nowadays, surgical treatment is more commonly used than conservation treatment.^[2,3]^ However, some complications can occur after the plate fixation (PF) in patients with DMCFs,^[4–13]^ including nonunion, infection, and other complications. Intramedullary fixation (IMF) is considered a better choice for these fractures.^[14]^ More and more studies showed that IMF for displaced fractures of the mid-shaft of the clavicle to be superior to PF treatment.^[13–17]^ However, the optimal method of this fracture remains a topic of debate. A significant body of literature has been devoted to the comparison of IMF and PF for DMCFs.^[3,14–20]^ RCTs comparing IMF and PF are conflicted as to which treatment is better than the other one. In addition, multiple authors have conducted meta-analyses comparing IMF and PF treatments.^[3,14–20]^ However, the results of the meta-analyses have been discordant in their findings. For example, a meta-analysis by Xiao et al^[19]^ and Zhu et al^[15]^ showed that IMF leads to a higher constant shoulder (CS) scores than PF treatment. However, Duan et al^[3]^ and Wang et al^[17]^ concluded that both IMF and PF treatments can achieve a similar CS scores. Debate continues in the literature and both treatments continue to be used frequently in practice.

The purposes of this study were: to perform a systematic review of overlapping meta-analyses comparing IMF and PF treatment for DMCFs, to help decision makers critically evaluate the current meta-analyses, and to propose a guide through the best available evidence.

## 2 Materials and methods

This systematic review was performed according to the guidelines of Preferred Reporting Items for Systematic Reviews and Meta-analyses.^[21]^ Ethical approval and informed patient consent were not required, as this study was a literature review and had no direct patient contact or influence on patient care.

### 2.1 Study search

We searched the Cochrane library, PubMed, and EMBASE. The following search terms were used: mid-shaft; clavicle; fracture; and meta-analyses or systematic review. The search was limited to articles written in English. All reviewed articles were then manually cross-referenced to ensure that all potential studies were included. The search was performed on February 2017.

### 2.2 Eligibility criteria

The study inclusion criteria were: meta-analyses including RCTs; meta-analyses comparing IMF with PF treatment for DMCFs; meta-analyses reported at least 1 variable. The exclusion criteria were: non-English language articles and meetings abstract. Full manuscripts were obtained for those studies that met both the inclusion and exclusion criteria. The references for each of these citations were then manually screened to ensure that no studies were missed.

### 2.3 Selection of meta-analyses

Two authors independently checked titles and abstracts from the searches to identify potentially eligible studies. The authors were not blinded to the names of original researchers, journals, or institutions. They independently retrieved and reviewed full-text articles for the purpose of applying eligibility criteria. When there were disagreements between authors, a consensus was reached through discussion or a third author was consulted.

### 2.4 Data extraction

Two authors independently extracted the data of each study. The following information of the meta-analyses was extracted: journal, date of literature search, search database, number of included trials, software use, and *I*^*2*^ statistic value. When there were disagreements between authors, a third author was consulted.

### 2.5 Quality assessment

Methodological quality for each included meta-analyses was assessed using the Oxford Levels of Evidence^[22]^ and Assessment of Multiple Systematic Review (AMSTAR).^[23]^ AMSTAR uses 11 items to assess which review methods are unbiased and are extensively applied. Both authors independently assessed methodological quality. Then the scores for every meta-analysis were calculated.

### 2.6 Assessment of heterogeneity

Heterogeneity information of each variable was extracted for the included studies. We explored whether the studies evaluated possible sources of heterogeneity across studies and whether the investigators performed a sensitivity analysis. According to the Cochrane Handbook, heterogeneity is considered not important between 0% and 40%; moderate between 30% and 60%; substantial between 50% and 90%, and considerable between 75% and 100%. Therefore, an *I*^*2*^ of <60% is accepted in this systematic review.

### 2.7 Application of Jadad decision algorithm

Four authors independently applied the Jadad algorithm^[24]^ and arrived at a consensus as to which of the systematic reviews provided the best currently available evidence. This methodology determines the source of discordance between systematic reviews from the following 6 reasons: clinical question, inclusion and exclusion criteria, data extraction, quality assessment, data pooling, and statistical analysis.

## 3 Results

### 3.1 Search results

**Figure 1:**
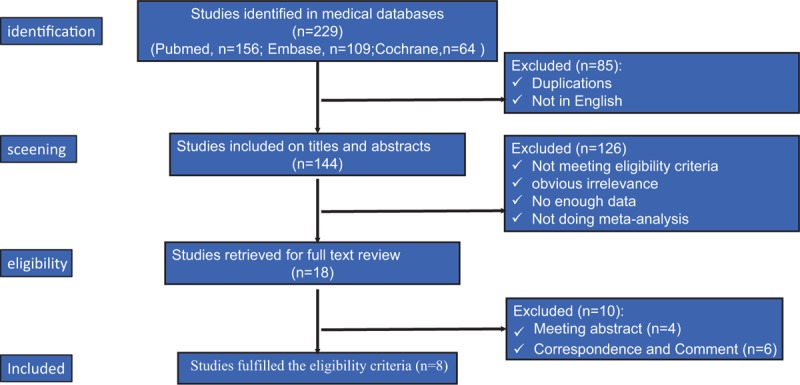
Flow diagram summarizing the selection process of meta-analyses.

A flow diagram that depicts the search process can be found in Figure 1. A total of 229 titles were found initially. Eight studies met the inclusion criteria. A general description of the characteristics of each meta-analysis is provided in Table 1. These studies were published between 2011 and 2016, and all 8 performed a meta-analysis.^[3,14–20]^ The number of primary studies varied widely from 2 in those studies published in 2011 to 7 for 1 study published in 2015 (Table 2).

**Table 1:**
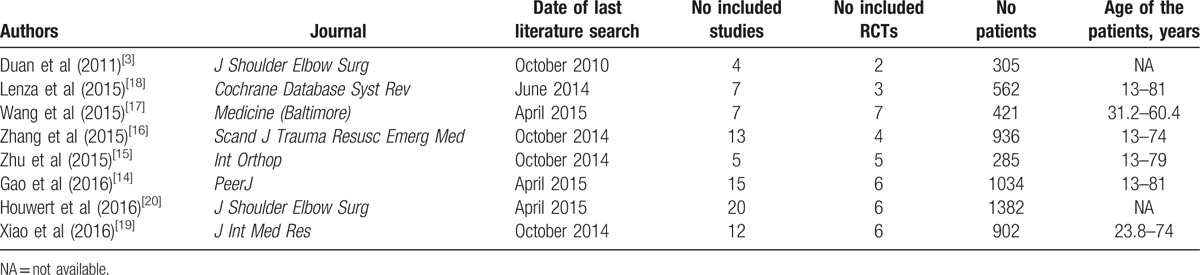
General description of the characteristics of each meta-analyses.

**Table 2:**
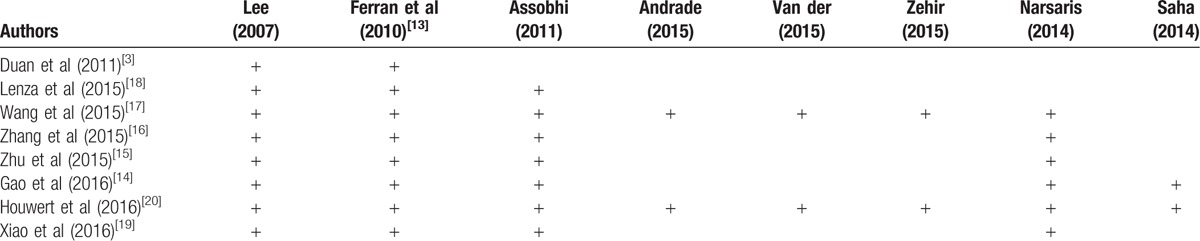
Primary studies included in meta-analyses.

### 3.2 Search methodology

Most studies comprehensively searched databases. All of the included studies searched Cochrane Library and PubMed. There was heterogeneity as to whether studies also included searches of Embase, OVID, and Google scholar. Table 3 gives details regarding search methodology used by each included study

**Table 3:**
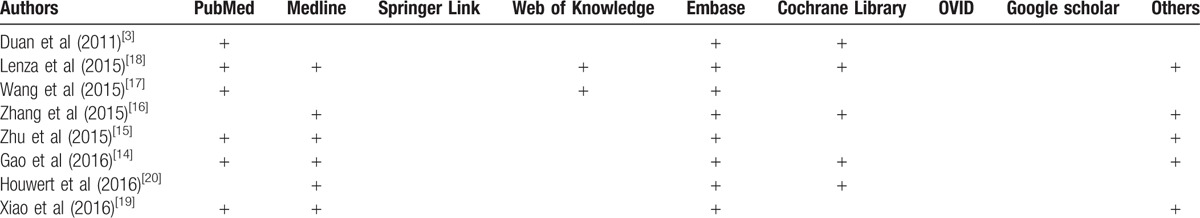
Databases used by each study in their literature searches.

### 3.3 Methodological quality

All studies included RCTs or observational study and were level II of evidence (Table 4). AMSTAR results for each question from each meta-analysis are shown in Table 5. AMSTAR scores varied from 6 to 9.

**Table 4:**
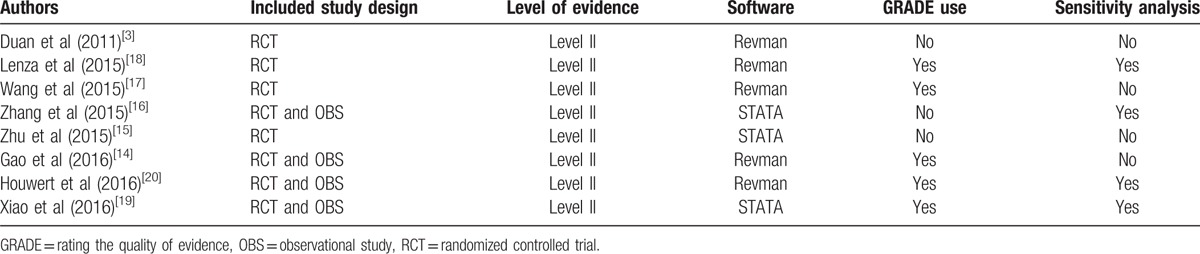
Methodological information for each included study.

**Table 5:**
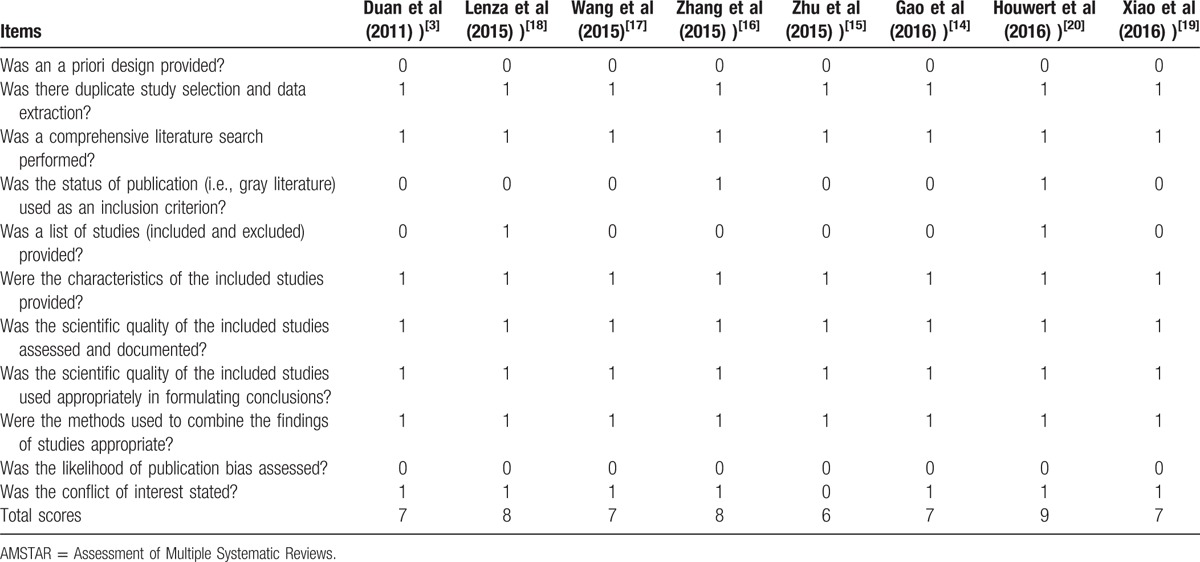
AMSTAR criteria for each included study.

### 3.4 Heterogeneity assessment

The *I*^*2*^ statistic value was calculated to assess study heterogeneity as a measure for determining the interstudy variability in all meta-analyses. Heterogeneity of each outcome was acceptable (<60%) in those meta-analyses pooled results (Table 6). Of the 8 meta-analyses, 4 meta-analyses conducted sensitivity analyses based on publication status or methodological quality (Table 4).

**Table 6:**
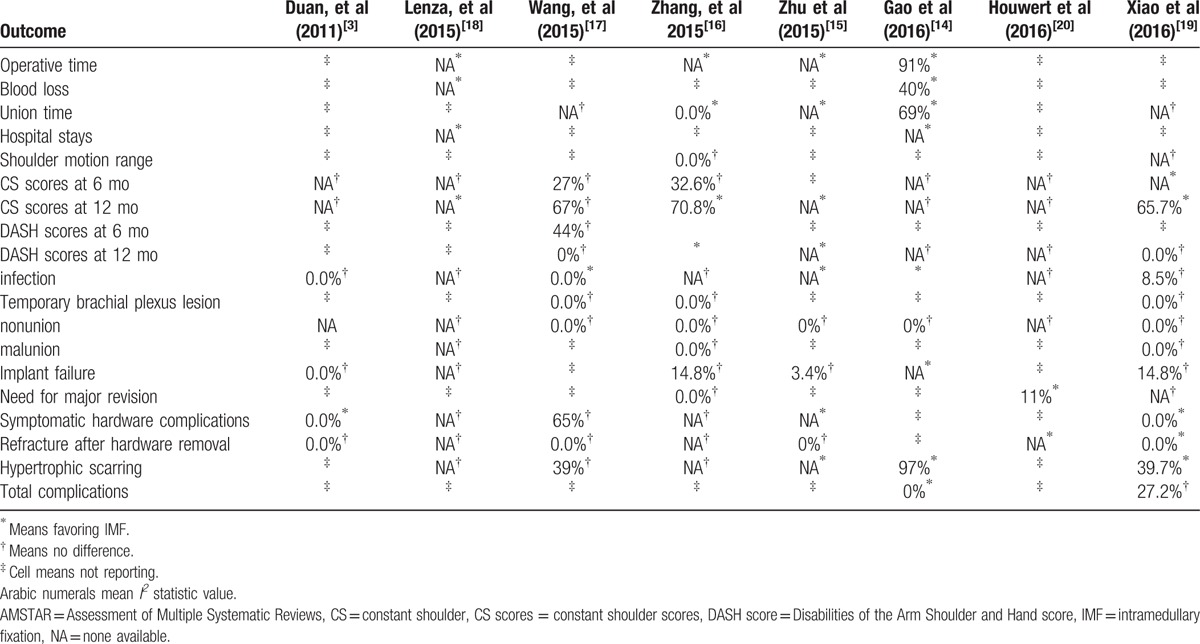
*I*^*2*^ statistic value of each variable in each meta-analysis.

### 3.5 Results of Jadad decision algorithm

**Figure 2:**
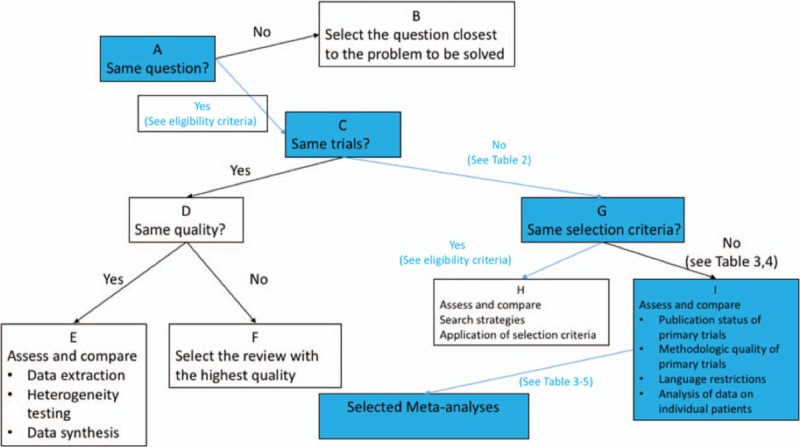
Flow diagram of Jadad decision algorithm.

The Jadad decision algorithm was applied to determine which of the included studies provided the best available evidence. The results of all included meta-analyses are summarized in Table 6. Given that the selection criteria were not accordant among included meta-analyses, the Jadad algorithm suggests that the highest-quality review should be selected based on the publication characteristics of the primary trials, the methodology of the primary trials, the language restrictions, and whether analysis of data on individual patients was included in the study. As a result, we selected a high-quality Cochrane review (Fig. 2). Study suggested that compared with PF, major reintervention and refracture after implant removal occurred more frequently after PF of noncomminuted, displaced DMCFs. No differences in terms of function and nonunion between PF and IMF were observed. Future research should focus on fracture selection for IMF and further improvement of plates and IM devices (Table 6).

## 4 Discussion

A number of RCTs have attempted to compare IMF and PF treatment with DMCFs. Systematic reviews or meta-analyses are considered the highest level of scientific evidence. Multiple meta-analyses have been published for the treatment of MCFs, they still reached different conclusions.^[3,14–20]^ These discordances complicate surgeons, patients, and policymakers, and our study has thus attempted to determine which of these studies represents the highest level of evidence on this topic to date.

Jadad et al^[24]^ summarized the potential sources of discordance among meta-analyses including the clinical question (population of patients, interventions, outcome measures, and setting), study selection and inclusion (selection criteria, application of selection criteria, and strategies used to search the literature), data extraction (methods used to measure outcomes, end points, and human error), assessment of study quality (methods used to assess quality, interpretations of quality assessments, and methods used to incorporate quality assessments in review), assessment of the ability to combine studies (statistical methods and clinical criteria used to judge the ability to combine studies), and statistical methods for data synthesis. Jadad et al also provided a decision tool (decision algorithm) to help decision-makers select from among discordant reviews. It is a useful tool for differentiating between overlapping reviews and was widely used as shown in the present study. Using the Jadad algorithm, 4 authors independently arrived at the conclusion that the review provided by Houwert et al^[20]^ provides the current highest level of evidence and it concludes that the differences between IMF treatment and PF treatment were not significant in function score, infection, and nonunion. But IMF treatment provides a lower rate of major revision and refracture hardware complications. With careful consideration of the relative advantages and disadvantages of each intervention and of patient preferences, IMF is a better choice.

There are numerous limitations to our study. First, our search strategy was limited by the exclusion of non-English literature that might have met our inclusion criteria, although we searched for as many meta-analyses as possible. Second, although only the meta-analyses exclusively including RCT design were assessed to ensure the high quality of this systematic review, all meta-analyses were level II evidence. And none of them was level I evidence.

## 5 Conclusions

In this systematic review of overlapping meta-analyses comparing operative and nonoperative treatment for displaced proximal humeral fractures involving the humeral neck suggested that according to current best available evidence, compared with IMF, major reintervention and refracture after implant removal occurred more frequently after PF of DMCFs. No differences in terms of function and nonunion between PF and IMF were observed. Future research should focus on fracture selection for IMF and further improvement of plates and IM devices.
